# Patient Portal Utilization Among Ethnically Diverse Low Income Older Adults: Observational Study

**DOI:** 10.2196/medinform.8026

**Published:** 2017-11-14

**Authors:** Thomas A Arcury, Sara A Quandt, Joanne C Sandberg, David P Miller Jr, Celine Latulipe, Xiaoyan Leng, Jenifer W Talton, Kathryn P Melius, Alden Smith, Alain G Bertoni

**Affiliations:** ^1^ Department of Family and Community Medicine Wake Forest School of Medicine Winston-Salem, NC United States; ^2^ Department of Epidemiology and Prevention Division of Public Health Sciences Wake Forest School of Medicine Winston-Salem, NC United States; ^3^ Department of Internal Medicine Wake Forest School of Medicine Winston-Salem, NC United States; ^4^ Department of Software and Information Systems University of North Carolina at Charlotte Charlotte, NC United States; ^5^ Department of Biostatistical Sciences Division of Public Health Sciences Wake Forest School of Medicine Winston-Salem, NC United States; ^6^ Greene County Health Care, Inc Snow Hill, NC United States

**Keywords:** electronic health records, electronic personal health information management, health disparities, aging, rural health

## Abstract

**Background:**

Patient portals can improve patient communication with providers, provide patients with greater health information access, and help improve patient decision making, if they are used. Because research on factors facilitating and limiting patient portal utilization has not been conceptually based, no leverage points have been indicated for improving utilization.

**Objective:**

The primary objective for this analysis was to use a conceptual framework to determine potentially modifiable factors affecting patient portal utilization by older adults (aged 55 years and older) who receive care at clinics that serve low income and ethnically diverse communities. The secondary objective was to delineate how patient portal utilization is associated with perceived usefulness and usability.

**Methods:**

Patients from one urban and two rural clinics serving low income patients were recruited and completed interviewer-administered questionnaires on patient portal utilization.

**Results:**

A total of 200 ethnically diverse patients completed questionnaires, of which 41 (20.5%) patients reported utilizing portals. Education, social support, and frequent Internet utilization improve the odds of patient portal utilization; receiving health care at a rural clinic decreases the odds of portal utilization.

**Conclusions:**

Leverage points to address disparities in patient portal utilization include providing training for older adults in patient portal utilization, involving spouses or other care partners in this training, and making information technology access available at public places in rural and urban communities.

## Introduction

### Background

Electronically supported forms of personal health information management is essential to the future of health care as these approaches facilitate improved health outcomes through improvement in health care quality and efficacy, decrease medical costs, and improve patient-physician communication [1-10]. For the potential of electronic personal health information management approaches to improve patients’ communication with their providers and for patients to access greater information that improves decision making, these patients must actually utilize electronic personal health information management applications. Patient portals are one approach to electronic personal health information management that has been discussed for its potential to benefit patients and to reduce health care costs. Patient portals enable secure messaging between patients and health care providers and give patients access to their personal health records [11-13]. Patient portals are a concern for health care providers, as the Centers for Medicare and Medicaid Services has mandated that providers achieve meaningful use of these portals by their patients. Patient portal utilization is especially important for older adults, as aging is associated with a growing number of health issues and disabilities, prescription medicines, and providers.

Research on patient portal utilization has included several approaches. First, analyses of electronic health (eHealth) records indicate a wide variation in the proportion of patients receiving the access code for their portal, activating their accounts, and actually utilizing their portals [14-17]. Although Gordon and Hornbrook [14] found that almost 80% of Kaiser Permanente older adult patients had enrolled in the health system’s patient portal, most other analyses have reported lower rates of patient portal utilization, with between 10% and 30% of patients activating or logging into their portals at least once and fewer than 10% being active portal users [15-17]. In addition to more women than men utilizing their patient portals, these secondary analyses consistently found that factors reflecting health disparities, including older age, lack of private health insurance, and minority group membership were related to lower patient portal utilization.

Cross-sectional surveys report similar low levels of patient portal utilization. Fewer than one-third of patients report having logged into their patient portal accounts in the past year [18,19]. Similar to analyses of electronic health records (EHRs), these primary surveys found that measures related to health disparities, including lower educational attainment, older age, minority group membership, and living in rural communities were associated with lower patient portal utilization [16,19]. Furthermore, Peacock and colleagues [18] found that health care providers were less likely to offer patient portal access to minority patients than white patients.

Analyses of factors affecting patients’ and caregivers’ portal utilization have used qualitative designs based on focus groups and individual in-depth interviews [20-24]. The need for technical assistance [21] and the lack of technological experience and access to technology [20], as well as a lack of facility with keyboards and screens [22] were discussed as barriers to patient portal utilization by patients and their caregivers. Limits to literacy and health literacy (the inability to read or understand information provided through the portal) also reduced patient portal utilization [22,23], as did concerns over information security [20,22,23]. Fear of losing a personal relationship with a health care provider and a preference for in-person communication with health care providers also curbed the desire to utilize a patient portal [20,21,23,24]. At the same time, poor existing relationships and communications with a health care provider increased the desire to utilize a patient portal [24]. Health care providers have also expressed concerns about patient portals, including uncertainties over increased workload [25,26], increased patient confusion, and alienating patients who do not utilize portals [26].

Finally, analyses examining the association of patient portal utilization on health outcomes have shown mixed effects. Portal utilization increases patient satisfaction and improves health services utilization [15,27-29], but data are insufficient for determining the effects of patient portal utilization on health outcomes [30]

The overall picture of patient portal utilization is that only a limited number of patients utilize these portals, with utilization decreasing with patient age. Several personal characteristics that reflect health disparities limit patient portal utilization. However, because much of this research is not conceptually based, no leverage points are indicated for improving patient portal utilization. Our primary aim for this analysis was to use a conceptual framework to determine potentially modifiable factors affecting patient portal utilization by older adults (those aged 55 years and older) who receive care at clinics that primarily serve low income, ethnically diverse communities. Our secondary aim was to delineate how patient portal utilization is associated with perceived usefulness and usability. This analysis is innovative in that it focuses on older adults who receive care from clinics concentrating on patients with limited resources, and it is conceptually based.

### Conceptual Framework

Our conceptual framework integrates concepts from Davis’ Technology Acceptance Model (TAM) [31] and the Person-Environmental Interaction Model [32] that have been influential for understanding users’ adoption of technologies [33-37]. On the basis of Fishbein and Azjen’s Theory or Reasoned Action and Theory of Planned Behavior [38], TAM posits that for acceptance of information technology (IT), belief leads to attitude leads to intention leads to behavior. Empirical results have demonstrated a parsimonious model containing two beliefs as the fundamental determinants of technology product utilization: perceived usefulness and perceived usability [35,39-41]. Perceived usefulness is the degree to which an individual believes that using a technology would enhance performance, whereas perceived usability is the degree to which an individual believes that using a technology would involve little effort [31].

**Figure 1:**
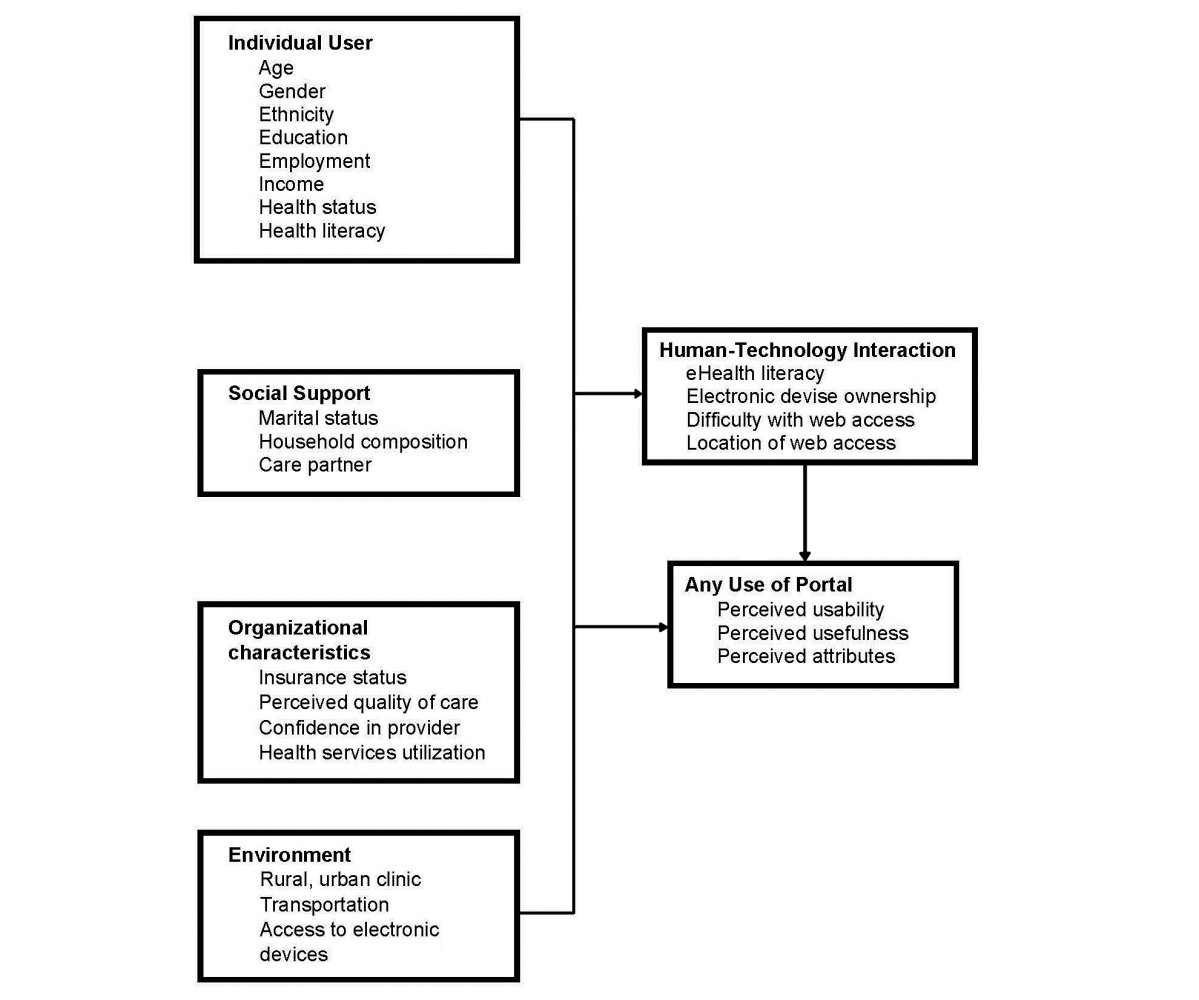
Conceptual framework.

Our framework addresses how key factors of (1) the individual user, (2) social support, (3) organizational characteristics, (4) environment, and (5) human-technology interaction influence the overall adoption process (Figure 1). Individual user characteristics such as education and health literacy are important for understanding patient portal utilization. Lower technology access and use are related to infrastructure barriers as well as demographic characteristics, including ethnicity (African American and Latino), advanced age, and lower income and education [42,43]. Older adults’ utilization of patient portals is influenced by their unique characteristics. For instance, age has been inversely related to technology interest and utilization [44,45], Internet utilization [46,47], and broadband access [42,43].

Successful patient portal adoption depends on two other key factors: social support and organizational characteristics. Older adults’ social network can be segmented into components (eg, spouse, children, and care partner), each having its own modalities to initiate and sustain patient portal utilization. Social support has been found to be predictive of health information technology utilization, although the findings are not consistent [37,48]. Technology utilization can also improve social support for older adults [49]. The health and communication information field still lacks a clear understanding between older users and the members of their social network to support IT behavior.

Some research suggests the importance of organizational characteristics in using health technologies. Satisfaction with medical care services and confidence in one’s health care provider are associated with technology acceptance [37,50,51]. Our rationale is that patient portal adoption will likely be embraced by satisfied patients. The extent to which older patients utilize patient portals likely depends on the frequency of care and having more confidence in one’s health care provider.

Users’ environmental contexts facilitate or impede patient portal adoption. Technology and contextual setting do not occupy separate domains but are intimately linked. Many individuals without Internet access make use of public resources such as libraries or community centers [52]; thus, public access sites serve to improve usage, contributing positively to patient portal acceptance among rural users.

The framework focuses on the human-technology interaction that is based on TAM. Human-technology interaction variables such as eHealth literacy and technology experience moderate the degree to which older adults utilize technology and their perception of its usability and usefulness [53].

## Methods

### Recruitment

Participants were recruited from urban and rural clinics that primarily serve low income patients. The urban clinic was the Outpatient Department Clinic, Department of Internal Medicine, Wake Forest Baptist Health, located in Winston-Salem, North Carolina. The outpatient department services ethnically diverse, low income, predominantly Medicare and Medicaid patients. Two members of Community Partners HealthNet were the rural clinics. Community Partners HealthNet is a health center–controlled network that was formed in 1999 to implement practice management systems for community health centers. Greene County Health Care Inc and West Caldwell Health Council Inc serve the rural areas of Greene and Caldwell Counties, North Carolina, respectively. Greene County Health Care Inc has six clinic locations. West Caldwell Health Council Inc has two clinic locations. The patient portal systems of the urban and rural clinics differed; the urban system had been established for several years and included a large number of features, whereas the system used in the rural clinics was new and had fewer features than those used by the urban clinic. Both systems were only available in English, making them difficult to use for patients from North Carolina’s growing Latino population, many of whom have limited English language skills.

Inclusion criteria were community-dwelling adults aged 55 years and older, who were being treated for a chronic disease (diabetes, hypertension, dyslipidemia, or cardiovascular disease), who spoke English or Spanish, and were in sufficiently good health to give informed consent and complete the series of interviews. The majority of patients were recruited using a three-step process. With the assistance of clinic staff, lists of patients who met the inclusion criteria were generated and shared with the project team members. Clinic staff reviewed these lists and indicated patients who might be willing to participate in the project. Potential participants were randomly selected from this list and sent letters introducing and describing the study. The letters indicated that patients were eligible to participate because they met the inclusion criteria. Follow-up phone calls were then made to further describe the study and to schedule interviews with those who received the letters. Additionally, Spanish-speaking participants were recruited as they came to one set of rural clinics. The data collector approached individuals fitting the inclusion criteria, described the study, and scheduled interviews for a later date. The study protocol was approved by the Wake Forest Baptist Health Institutional Review Board, and all participants provided signed informed consent.

**Figure 2:**
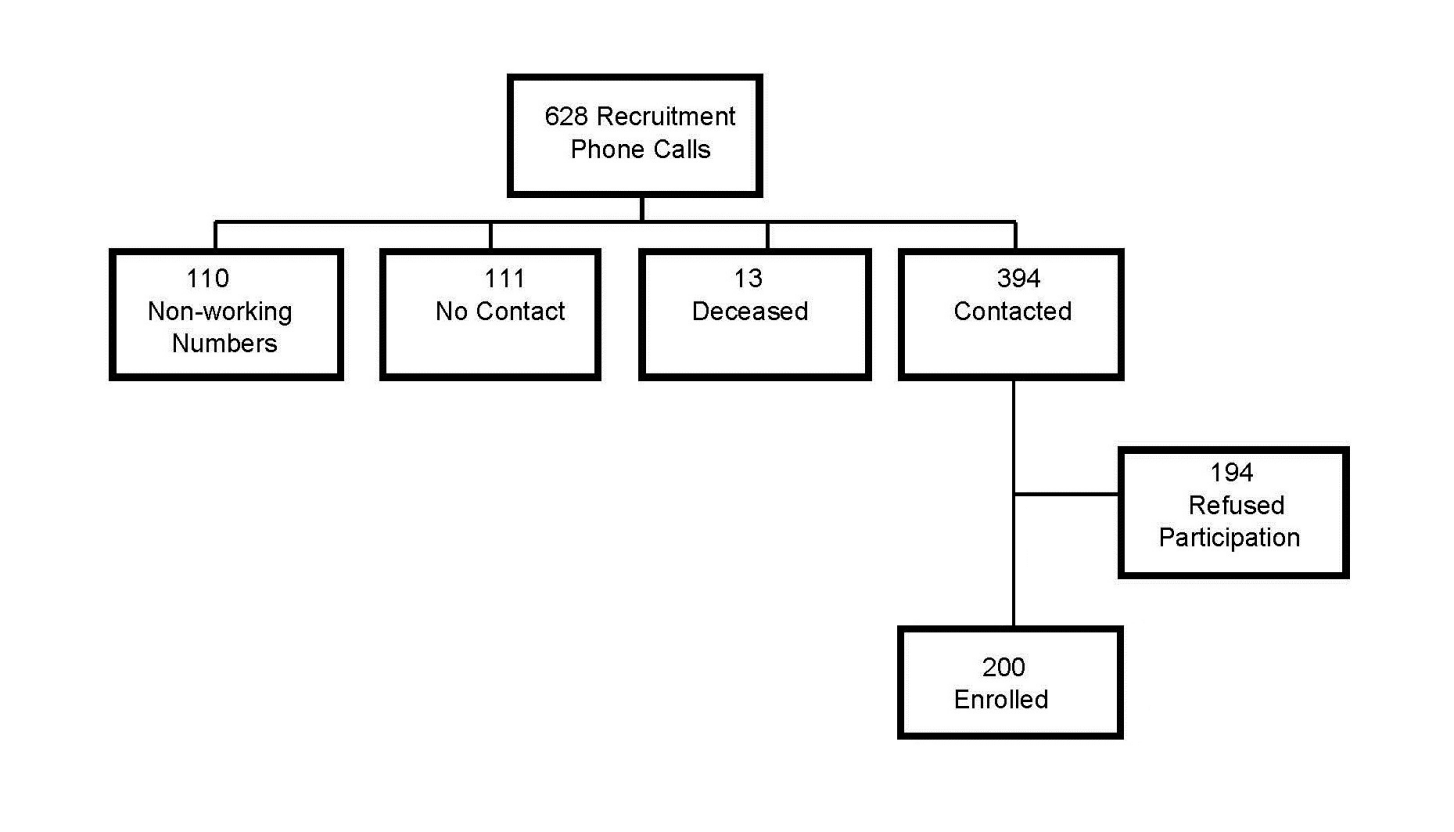
Patient recruitment flowchart.

The patient sample included 200 African American, white, Latino, and other older adult patients who completed baseline interviews (Table 1). Data collectors attempted to contact 628 patients by letter or in person, with a follow-up telephone call (Figure 2). Of the 628 attempted contacts, 110 had a nonworking telephone number, 111 could not be contacted by telephone, 13 were deceased, and 394 were contacted for a contact rate of 62.7% (394/628). Of the 394 who were contacted, 194 refused to participate, for a refusal rate of 49.2% (194/394); and 200 participants were successfully enrolled and completed the interviews, for an overall participation rate of 31.8% (200/628). Common reasons for refusing included not being interested (90 individuals), being too busy (33 individuals), being too ill (29 individuals), caring for a family member (5 individuals), and having changed location (4 individuals). Those who refused to participate were equally divided among women and men. However, more white (42.3%; 82/194) than African American (22.2%; 43/194) or Latino (0.0%) patients refused to participate, and more urban clinic (66.1%; 128/194) than rural clinic (28.9%; 56/194) patients refused to participate.

### Data Collection

The patient questionnaire included items eliciting information on personal characteristics such as age, race, marital status, and educational attainment; social interaction and social support; health characteristics such as chronic conditions, cognitive status, use of prescription medicines, health-related quality of life, health literacy; Internet and other modes of technology access; access to health care; and orientation and utilization of electronic health information resources such as a patient portal.

Questionnaires were always administered in person by trained interviewers, usually at the participants’ homes or at the clinic where they received medical care. Interviews were completed from November 2014 to May 2016. The interview generally took 1 hour to complete and ranged in length from 45 min to 2 hours. Participants were given an incentive of US $20 for completing the interview. Research Electronic Data Capture [54], a secured web-based system, was used to record interview data.

**Table 1:** Sample size by gender and ethnicity.

Ethnicity	Gender		
	Female	Male	Total
White	43	37	80
African American	53	37	90
Latino	17	9	26
Other	3	1	4
Total	116	84	200

### Measures

Patient portal utilization had the values of ever used versus never used based on patient self-report. Measures of the number of patient portal features used, patient portal positive perceptions, and utilization frequency are included for participants who were ever-users. Participants were asked whether they used 7 patient portal features (send a message to your doctor or nurse, refill prescriptions, view lab or test results, make or change an appointment, request a referral, find information about a health issue, and other). The number of features used were summed and placed in the categories 3 or less and 4 to 7; frequencies of use for the specific features are reported in Multimedia Appendix 1 **.** The use frequency item had the values several times a week, several times a month, less than once a month, a few times a year, and never. Responses were placed in the categories at least once a month (once a month, several times a month, and several times a week), and less than one a month (less than once a month, a few times a year, and never). Patient portal positive perceptions included 16 statements adapted from the Technology Acceptance Scale developed by Gardner and Amoroso [55] (eg, “Using my patient portal can enable me to accomplish tasks more quickly” and “Using my patient portal can make it easier to do my tasks.”). Agreement with statements was summed, with scores ranging from 0 to 16. Scores were placed in the categories 0 to 12 and 13 to 16 for analysis. Frequencies for the specific positive perceptions are reported in the Multimedia Appendix 1.

Individual user characteristics included age (in the categories 55-59 years, 60-64 years, 65-69 years, and 70 years and older), gender, ethnicity (in the categories white and minority), and education (in the categories high school or less and greater than high school). Employment had the values of not employed or employed (whether part- or full-time). Poverty level was based on the total household income divided by the total number of residents and adjusted for the year in which the data were collected [56]. Poverty level was placed in the categories less than 100% of poverty level, 100% to 200% of poverty level, and greater than 200% of poverty level. Health status was measured with the Charlson Comorbidity Index [57] that includes the self-reported diagnosis of 18 different chronic conditions. Participants were classified as having fewer than 5 versus 5 or more chronic conditions. All of the participants had at least one chronic condition (diabetes, hypertension, dyslipidemia, or cardiovascular disease) that required lab tests or ongoing medication. The categories used differentiate the ill from the very ill. Health literacy was measured with the Newest Vital Sign (NVS) [58]. This scale had a range of 0 to 6 and had a Cronbach alpha of .82. On the basis of the recommendation of the scale developers, those with a score of 4 to 6 were considered to have adequate health literacy, and those with a score of 0 to 3 were considered to have inadequate health literacy.

Three social support measures were included. Marital status had the values currently married and not currently married. Household composition had the values live alone, live with a spouse and no others, live with spouse and adult child, and other. Having a care partner was dichotomous. A care partner was defined for the participants as, “someone who helps (you) with activities and questions about (your) health. These activities and questions include simple things, like reminding you to take your prescription and about an upcoming doctor’s appointment, or finding information about something the doctor has told you; they can include more substantial assistance, like taking you to an appointment, helping you take your medicine, and helping you exercise; and they can include personal assistance, like helping you get dressed and bathe. Those who help you can be your spouse, brothers or sisters, children, or friends. The person who helps you may live with you, but they can also live in another house. They might even live in another town or city and help you by phone or the Internet.”

Health insurance, the first organizational measure, had the values private insurance, government insurance (eg, Medicare, Medicaid, and Veterans Administration), and no insurance. Difficulty in contacting the medical office during regular hours was dichotomous. Difficulty in contacting the medical office after regular hours and whether the medical office has night or weekend office hours had the values no, yes, and don’t know.

Whether the patient was recruited from a rural (Greene County Health Care Inc or West Caldwell Health Council Inc) or urban (Wake Forest Baptist Health Out Patient Department) clinic was the first environmental measure. Difficulty in accessing email in the county had the values difficult, easy, and don’t know.

Human-technology interaction measures included whether the patient sends and receives emails. eHealth literacy was measured with the 8-item eHealth Literacy Scale (eHEALS) [59,60] that had a range of 8 to 40, an overall mean of 22.7, an SD of 9.5, and a Cronbach alpha of .95. Access to e-devices and Internet at home (including desktop, laptop, tablet, and mobile phone) was dichotomous. Number of e-devices and Internet at home categories had the values of 0, 1, and 2 or more. Internet use frequency had the values less than once a day and at least once a day. Stress experienced when using computer was based on the item, “How much stress do you feel when using a computer?” which had the response categories no stress at all to very much stress; responses were placed in the categories no stress (no stress at all) and some stress (low stress, moderate stress, much stress, and very much stress).

### Statistical Analysis

All analyses were performed using SAS 9.4 (SAS Institute Inc). Personal characteristics were compared between patients who ever used a patient portal and those who never used a patient portal using chi-square test for categorical variables and student *t* test for continuous variables. A logistic regression model was used to examine association between personal characteristics and patient portal utilization. Factors in each of the conceptual framework key domains (the individual user, social support, organizational characteristics, environment, and human technology) that had a statistically significance associations with patient portal utilization based on chi-square tests and *t* tests were included in the logistic regression model. No organizational characteristics had a statistically significant bivariate association with patient portal utilization; the health insurance variable was selected to represent this key factor in the logistic regression model. Odds ratios (OR) with the corresponding 95% CI were estimated for each characteristic. Furthermore, associations between participants’ patient portal utilization and perceived usefulness were examined in terms of factors that remained statistically significant in the multivariable logistic regression model using chi-square test. All tests were two-sided and performed at a significance level of .05.

## Results

### Factors Associated With Ever Using a Patient Portal

Participant characteristics, organized by domain within the conceptual framework and their association with patient portal utilization are reported in Table 2. A total of 41 (20.5%) participants reported utilizing their patient portals. Patient portal utilization did not differ by participant age or gender. More white participants (37.5%) than minority participants (9.2%), and more with greater than a high school education (47.1%) than with a high school education or less (6.2%) had utilized their patient portal. Employment was not associated with patient portal utilization but poverty level was: 9.0% of those below the poverty level, 25.3% of those at 100% to 200 % of the poverty level, and 46.9% of those above 200% of the poverty level had utilized their patient portal. Those with worse health, as indicated by a Charlson Comorbidity Index of 5 or more, utilized their patient portal more (25.4%) than did those with a score below 5 (9.7%). Over half (50.9%) of those with adequate health literacy (NVS score of 4-6) utilized their patient portal, whereas 9.6% of those with inadequate health literacy utilized their patient portal.

Participants who were married (29.8%) and lived only with their spouse (34.5%) utilized their portal more than those not married (13.8%) and who had other household composition. Identifying a care partner was not associated with patient portal utilization. Type of health insurance, difficulty with contacting the medical office during or after regular hours, and whether the medical office had night or weekend office hours were not associated with portal utilization. Receiving care at an urban (30.0%) rather than a rural (6.3%) clinic was associated with portal utilization. Difficulty of accessing email in the county was not associated with patient portal utilization.

Greater eHealth literacy, as measured by the eHEALS scale, was associated with patient portal utilization; the mean eHEALS value for the entire sample was 22.7 (SD 9.5), whereas it was 31.7 (SD 6.5) for portal users and 20.2 (SD 8.7) for those not utilizing their portal (*P*<.001). A far greater percentage of participants who send and receive email (51.3%) utilized a patient portal than those who did not use email (0.8%). Those with access to e-devices and Internet in their homes (33.9% vs 1.2%), those with 2 or more e-devices in their home (36.8% vs 12.2% with 1 device and 0% with no device), who use the Internet at least once a day (47.5% vs 8.6%), and who experience no stress when using a computer (50.0% vs 11.3% who experience at least some stress) were more likely to utilize their patient portal.

**Table 2:** Participant characteristics and their association with patient portal utilization.

Participant characteristics			Overall (N=200) n (%)	Ever used patient portal (n=41) n (%)	*P* value
**Individual user**					
	**Age in years**				.71
		55-59	55 (27.5)	11 (20.0)	
		60-64	63 (31.5)	15 (23.8)	
		65-69	52 (26.0)	11 (21.2)	
		70 and older	30 (15.0)	4 (13.3)	
	**Gender**				.32
		Male	84 (42.0)	20 (23.8)	
		Female	116 (58.0)	21 (18.1)	
	**Ethnicity**				<.001
		White	80 (40.0)	30 (37.5)	
		Minority	120 (60.0)	11 (9.2)	
	**Education**				<.001
		High school or less	130 (65.0)	8 (6.2)	
		Greater than high school	70 (35.0)	33 (47.1)	
	**Employment**				.34
		Not employed	160 (80.0)	35 (21.9)	
		Employed (part-time or full-time)	40 (20.0)	6 (15.0)	
	**Poverty level**				<.001
		Less than 100% of poverty level	89 (46.4)	8 (9.0)	
		100-200% of poverty level	71 (37.0)	18 (25.3)	
		Greater than 200% of poverty level	32 (16.6)	15 (46.9)	
	**Charlson Comorbidity Index**				.03
		Fewer than 5	62 (31.0)	6 (9.7)	
		5 or more	138 (69.0)	35 (25.4)	
	**Newest Vital Sign**				<.001
		Adequate literacy (score of 4-6)	53 (28.0)	27 (50.9)	
		Inadequate literacy (0-3)	136 (72.0)	13 (9.6)	
**Social support**					
	**Marital status**				<.01
		Currently married	84 (42.0)	25 (29.8)	
		Not currently married	116 (58.0)	16 (13.8)	
	**Household composition**				.02
		Live alone	70 (35.0)	12 (17.1)	
		Live with spouse (no other residents)	55 (27.5)	19 (34.5)	
		Live with spouse and adult child	25 (12.5)	4 (16.0)	
		Other	50 (25.0)	6 (12.0)	
	**Care partner**				.88
		No	76 (38.0)	16 (21.1)	
		Yes	124 (62.0)	25 (20.2)	
					
**Organizational characteristics**					
	**Health insurance**				.22
		Private insurance	54 (27.0)	15 (27.8)	
		Government insurance	121 (60.5)	23 (19.1)	
		None	25 (12.5)	3 (12.0)	
	**Difficulty in contacting the medical office during regular hours**				.67
		No	126 (63.0)	27 (21.4)	
		Yes	74 (37.0)	14 (18.9)	
	**Difficulty in contacting the medical office after regular hours**				.08
		No	57 (28.5)	13 (22.8)	
		Yes	65 (32.5)	18 (27.7)	
		Don’t know	78 (39.0)	10 (12.8)	
	**Medical office has night or weekend office hours**				.16
		No	99 (49.5)	25 (25.3)	
		Yes	50 (25.0)	6 (12.0)	
		Don’t know	51 (25.5)	10 (19.6)	
**Environment**					
	**Clinic**				<.001
		Rural	80 (40.0)	5 (6.3)	
		Urban	120 (60.0)	36 (30.0)	
	**Difficulty in accessing email in the county**				.15
		Difficult (very difficult, difficult, neutral)	32 (16.0)	7 (21.9)	
		Easy (easy, very easy)	133 (66.5)	31 (23.3)	
		Don’t know	35 (17.5)	3 (8.6)	
**Human technology**					
	**Send and receive email**				<.001
		No	122 (61.0)	1 (0.8)	
		Yes	78 (39.0)	40 (51.3)	
	**Access to e-devices and Internet at home**				<.001
		No	82 (41.0)	1 (1.2)	
		Yes	118 (59.0)	40 (33.9)	
	**Number of e-devices and Internet at home**				<.001
		0	56 (28.0)	0	
		1	49 (24.5)	6 (12.2)	
		2 or more	95 (47.5)	35 (36.8)	
	**Internet use frequency**				<.001
		Less than once a day	139 (69.5)	12 (8.6)	
		At least once a day	61 (30.5)	29 (47.5)	
	**Stress experienced when on computer**				<.001
		No stress	48 (24.1)	24 (50.0)	
		Some stress	151 (75.9)	17 (11.3)	

Multivariate analysis addressing patient portal utilization was conducted using measures from each framework domain (Table 3). Measures selected for this analysis were significant in the bivariate analysis but not collinear with other measures. The organizational characteristic measure health insurance was included in the multivariate analysis even though it did not have a significant bivariate association to have this key factor included. The human-technology measures were all highly intercorrelated; Internet use frequency was selected over other measures (eg, eHEALS score) because asking a patient whether they used the Internet at least once a day is a procedure that a health care provider promoting patient portal utilization could easily accomplish during a patient visit.

Several personal characteristics were not significantly associated with patient portal utilization in the multivariate analysis, including ethnicity, health status or Charlson Index, and type of health insurance. Other personal characteristics sustained statistically significant associations with patient portal utilization. Those with greater than a high school education had greater odds of patient portal utilization (Odds ratio [OR] 5.75, 95% CI 1.94-17.04). Those who were not currently married had lesser odds of patient portal utilization (OR 0.17, 95% CI 0.06-0.52). Receiving care at an urban clinic greatly increased the odds of patient portal utilization (OR 12.21, 95% CI 3.05-48.87). Finally, using the Internet at least daily increased the odds of patient portal utilization (OR 7.08, 95% CI 2.55-19.67).

### Utilization of Patient Portal Features

Of 41 participants who utilized their patient portal, almost half (48.8%) utilized at least four portal features (Table 4), such as sending a message to a doctor or nurse or refilling prescriptions. Most (70.7%) users utilized their patient portal at least once a month. Most (61.0%) users had positive perceptions of most patient portal attributes. Users who were not currently married more often (68.8%) utilized at least four portal features than those who were currently married (36.0%; Table 5). Users who received care at urban clinics more often (55.6%) utilized at least four portal features than did those who received care at rural clinics (0.0%). Frequency of portal utilization did not differ among users for the characteristics considered (Table 6). Positive perceptions of patient portal attributes were greater among those who received care at urban clinics (69.4%) than at rural clinics (0.0%) (Table 7).

**Table 3:** Logistic regression models of patient portal utilization.

Personal characteristics	Ever used patient portal	
	Odds ratio (95% CI)	*P* value
Minority versus white (white as reference)	0.55 (0.19-1.60)	.27
Greater than high school versus high school or less	5.75 (1.94-17.04)	<.01
Charlson Index: 5 or more versus fewer than 5	2.43 (0.65-9.13)	.18
Not currently married versus married	0.17 (0.06-0.52)	.001
Private insurance versus none	0.73 (0.11-5.07)	.86
Government insurance versus none	0.61 (0.09-4.10)	
Urban versus rural clinic	12.21 (3.05-48.87)	<.01
Internet frequency: at least once a day versus less than once a day	7.08 (2.55-19.67)	<.01

**Table 4:** Participant patient portal utilization and perceived usefulness.

Patient portal utilization and perceived usefulness		n (%)
**Patient portal features utilized**		
	3 or less	21 (51.2)
	4 to 7	20 (48.8)
**Utilization frequency**		
	Less than once a month	12 (29.3)
	At least once a month	29 (70.7)
**Patient portal positive perceptions**		
	12 or less	16 (39.0)
	13 to 16	25 (61.0)

**Table 5:** Factors associated with patient portal feature utilization.

Participant characteristics		Patient portal features used		
		3 or less, n (%)	4 to 7, n (%)	*P* value
**Education**				.70
	High school or less	5 (62.5)	3 (37.5)	
	Greater than high school	16 (48.5)	17 (51.5)	
**Marital status**				.04
	Currently married	16 (64.0)	9 (36.0)	
	Not currently married	5 (31.2)	11 (68.8)	
**Clinic**				.03
	Rural	5 (100.0)	0 (0.0)	
	Urban	16 (44.4)	20 (55.6)	
**Frequency of Internet use**				.20
	Less than once per day	8 (66.7)	4 (33.3)	
	At least once per day	13 (44.8)	16(55.2)	

**Table 6:** Factors associated with patient portal utilization frequency.

Participant characteristics		Use frequency		
		Less than once per month n (%)	At least once per month n (%)	*P* value
**Education**				>.99
	High school or less	6 (75.0)	2 (25.0)	
	Greater than high school	23 (69.7)	10 (30.3)	
**Marital status**				.73
	Currently married	17 (68.0)	8 (32.0)	
	Not currently married	12 (75.0)	4 (25.0)	
**Clinic**				.13
	Rural	2 (40.0)	3 (60.0)	
	Urban	27 (75.0)	9 (25.0)	
**Frequency of Internet use**				.28
	Less than once per day	7 (58.3)	5 (41.7)	
	At least once per day	22 (75.9)	7 (24.1)	

**Table 7:** Factors associated with patient portal positive perceptions.

Participant characteristics		Patient portal positive perceptions		
		12 or less, n (%)	13 to 16, n (%)	*P* value
**Education**				.12
	High school or less	1 (12.5)	7 (87.5)	
	Greater than high school	15 (45.5)	18 (54.56	
**Marital status**				.41
	Currently married	11 (44.0)	14 (56.0)	
	Not currently married	5 (31.3)	11 (68.8)	
**Clinic**				<.01
	Rural	5 (100.0)	0 (0.0)	
	Urban	11 (30.6)	25 (69.4)	
**Frequency of Internet use**				.48
	Less than once per day	6 (50.0)	6 (50.0)	
	At least once per day	10 (34.5)	19 (65.5)	

## Discussion

### Principal Findings

Only a moderate proportion of patients participating in this study (20.5%) reported utilizing their patient portal when compared with the results of other primary surveys [18,19] and to the analyses of electronic records [14-17]. Patient portal utilization among patients participating in our survey was not comparable to the 80% utilization among older patients in the California Kaiser Permanente system [14]. Contrary to other research, we did not find differences in portal utilization by age or gender among our participating patients. The lack of variation in portal utilization by age may reflect the relatively “young” patients who participated, with few over the age of 75 years. Having insurance, whether private or government, was not associated with patient portal utilization as indicated by Ancker and colleagues [15], perhaps because so many of our participants had government insurance (Medicare) and so few had no insurance. We did find differences in portal utilization by ethnicity, education, poverty level, and rurality; those who are minority, have lower income and education, and are rural utilize patient portals less. Differences in participant portal utilization by these characteristics reflect disparities. Even in this relatively low income population, a social gradient in utilization is apparent [61].

As qualitative analyses suggested, familiarity and use of technology were associated with greater patient portal utilization [20-23]. Those patients with limited access to electronic devices in their homes, who seldom used electronic devices, and who experienced stress when using computers were less likely to have utilized a patient portal. Health literacy and eHealth literacy were associated with patient portal utilization in the bivariate analysis. Health literacy is important in mediating the ability of patients to access their patient portals and to interpret the medical information on their portals, which may influence willingness to use [62,63]. Tieu et al [22,23] report that health literacy was an important factor in their utilization of their patient portals.

Our multivariate analysis delineates factors that are important to patient portal utilization, including education, having a spouse (the most common form of social support), and frequent use of the Internet. It reveals that receiving health care in rural communities is associated with limited patient portal utilization; rural communities have less Internet access [64].

The results for patient portal usefulness and usability are somewhat tautological, that is, those who utilize their patient portal at all generally utilize several features. Most utilize their portal at least several times per month, and they generally have positive perceptions of their portal. Few factors differentiate levels of utilization and perceived usefulness, with the exception of rurality. Rural older adults utilize fewer portal features and have fewer positive perceptions; this reflects the fewer features available. The portal available through the rural clinics is not as robust as that available through the urban clinic. Future research needs to include a clear understanding of patient portal sophistication when comparing utilization and usability among users.

This conceptually based analysis indicates leverage points for improving patient portal utilization in health disparities communities, particularly minority and rural communities, in which a greater portion of members have chronic conditions and less access to health care than in the general population. Rurality limits patient portal utilization. Improving Internet connectively across all rural communities would improve patients’ ability to connect their patient portals [64]. Of course, improving Internet connectivity in rural communities would lead to other social and economic benefits [65]. Such infrastructure development for rural communities is not novel; the Rural Electrification Administration [66] provided similar rural infrastructure development in the 1930s.

Ethnicity does not remain a significant limitation to patient portal utilization when we control for other factors. Education remains a significant advantage for patient portal utilization. The complement to education, familiarity and regular use of Internet applications also improves patient portal utilization as documented in this and other analyses [20-23]. Community programs that provide Internet training and access for residents, particularly older adults and members of vulnerable minority populations, will help improve their patient portal utilization. Libraries have become Internet user hubs [67], including those in rural communities, and provide one institution that could be recruited for this purpose. Most communities, including rural communities, have facilities in addition to libraries, such as senior centers, congregate meal sites, recreational centers, and churches, in which computers can be located for Internet training and access; for example, WinstonNET has provided such training in Winston-Salem, North Carolina, for over 15 years [68]. Ensuring that residents know that they can access the Internet at diverse locations in their communities can support patient portal utilization. Making patient portals available in the language in which the patient is most comfortable would also improve utilization.

Familiarity and use of the Internet also provides an easy indicator for clinicians when assessing patients for potential patient portal utilization. Asking patients how often they use the Internet or email is more parsimonious than any multi-item scale for predicting patient portal adoption. Finally, involving a patients’ social support, particularly a spouse, can improve patient portal adoption and utilization. Sarkar and Bates [69] note the importance of involving family members or other “care partners” in training older adults to utilize patient portals. Such social support can be extremely important for attaining these skills within health disparities communities. Helping patients involve family members and share health information can improve the role of patient portals in the provision of patient-centered care. However, more research is needed to ensure processes that protect patients’ sense of privacy and autonomy [70].

Health care organizations must maintain the position that patient portals are a crucial mechanism to improve patient health and well-being, and they must convey this to patients to ensure utilization. Since this research began, the standards for patient portal meaningful use have deteriorated substantially, simply having a portal and having utilization by a single patient constitutes compliance. Some organizations do see the potential for patients. For example, since we began writing this paper, one organization, Wake Forest Baptist Health, has initiated a marketing campaign to increase patient portal utilization. This campaign includes billboards, radio advertisements, and television advertisements of very high production values.

### Limitations

This research should be evaluated within its limitations. The sample was drawn from patients receiving care at three sets of clinics (one urban and two rural). The participation rate was limited. These factors limit the generalizability of the results. The urban and rural clinics differed in the features available in their patient portals and in the time that they had been established before data collection was conducted. These factors could affect the differences between patients in their patient portal utilization. The key outcome measure, patient portal use, is based on self-reported use rather than capturing actual use using EHRs. This could limit the validity of this measure. At the same time, this survey did recruit a large, multi-ethnic, low income sample that included rural and urban patients.

### Conclusions

This analysis found that variation in patient portal utilization reflects disparities, even in low income patient populations. Our conceptual approach allows the delineation of leverage points to address these disparities that can be addressed by public policy and health policy, specifically the need to provide training for older adults in the utilization of IT in general and specifically of patient portals, involving family members (spouses) or other care partners in this training [69] and making IT widely (geographically) available at public places in rural as well as urban communities [67]. Future research should examine whether these strategies successfully lead to higher rates of portal use by vulnerable older adults.

## Appendix

Multimedia Appendix 1 Supplemental tables.

## Abbreviations

eHealth: electronic health
eHEALS: eHealth Literacy Scale
EHR: electronic health record
IT: information technology
NVS: Newest Vital Sign
OR: odds ratio
TAM: technology acceptance model
