# Supplementary material for: Patient Portal Utilization Among Ethnically Diverse Low Income Older Adults: Observational Study
Source: JMIR Med Inform. 2017 Nov 14;5(4):e47. doi: 10.2196/medinform.8026 (PMC5705857; doi:10.2196/medinform.8026)
Supplement: Multimedia Appendix 1 [file medinform_v5i4e47_app1.pdf]

Patient Portal Utilization among Ethnically Diverse Low Income Older Adults

Supplemental Tables

Patient Portal Features Used by Participants Who Use Their Patient Portal (n = 41)

	n	%
**Patient Portal Feature Used**		
Send a message to your doctor or nurse	28	68.3
Refill prescriptions	18	43.9
View lab or test results	37	90.2
Make or change an appointment	21	51.2
Request a referral	7	17.1
Find information about a health issue	17	41.5
Other	6	14.6
**Number of Patient Portal Feature Used**		
1	8	19.5
2	6	14.6
3	7	17.1
4	12	29.3
5 or more	8	19.5

Perceptions of the Patient Portal Among Participants Who Use Their Patient Portal (n = 41)

	n	%
Perceptions of Patient Portal		
It was easy to register to use my patient portal.	36	87.80
It is easy logging into my patient portal.	38	92.68
My medical information on my patient portal is accurate and up-to-date.	34	82.93
It is easy to navigate to different reports or lists and other features of my patient portal.	32	78.05
The information on my patient portal is easy to understand.	34	82.93
Using my patient portal makes me feel more connected with my doctor.	33	80.49
My communication with my doctor's office has been better since I've been using my patient portal.	25	60.98
Overall I'm satisfied with my patient portal.	33	80.49
Using my patient portal can enable me to accomplish tasks more quickly	30	73.17
Using my patient portal can make it easier to do my tasks	29	70.73
Learning to use my patient portal is easy for me	35	85.37
My interaction with my patient portal is clear and understandable	36	87.80
I always try to use my patient portal to do a task whenever it has a feature to help me perform it.	27	65.85
I expect my use of my patient portal to continue in the future.	38	92.68
Using my patient portal can take up too much of my time when performing many tasks.	4	9.76
I have a great deal of experience using my patient portal.	24	58.54
Total Number of Positive Responses		
3	1	2.44
4	2	4.88
7	1	2.44
10	5	12.20
11	4	9.76
12	3	7.32
13	2	4.88
14	8	19.51
15	5	12.20
16	10	24.39
